# Exploratory Full-Field Strain Analysis of Regenerated Bone Tissue from Osteoinductive Biomaterials

**DOI:** 10.3390/ma13010168

**Published:** 2020-01-01

**Authors:** Marta Peña Fernández, Cameron Black, Jon Dawson, David Gibbs, Janos Kanczler, Richard O. C. Oreffo, Gianluca Tozzi

**Affiliations:** 1 School of Mechanical and Design Engineering, University of Portsmouth, Portsmouth PO1 3DJ, UK; marta.pena-fernandez@port.ac.uk; 2 Bone & Joint Research Group, Centre for Human Development Stem Cells and Regeneration, Faculty of Medicine, University of Southampton, Southampton SO16 6YD, UK; CBlack@fitzpatrickreferrals.co.uk (C.B.); jid@soton.ac.uk (J.D.); d.gibbs@soton.ac.uk (D.G.); J.Kanczler@soton.ac.uk (J.K.); Richard.Oreffo@soton.ac.uk (R.O.C.O.); 3 School of Maritime Science and Engineering, Solent University, Southampton SO14 0YN, UK

**Keywords:** bone regeneration, biomaterials, *in situ* mechanics, microCT, digital volume correlation

## Abstract

Biomaterials for bone regeneration are constantly under development, and their application in critical-sized defects represents a promising alternative to bone grafting techniques. However, the ability of all these materials to produce bone mechanically comparable with the native tissue remains unclear. This study aims to explore the full-field strain evolution in newly formed bone tissue produced in vivo by different osteoinductive strategies, including delivery systems for BMP-2 release. In situ high-resolution X-ray micro-computed tomography (microCT) and digital volume correlation (DVC) were used to qualitatively assess the micromechanics of regenerated bone tissue. Local strain in the tissue was evaluated in relation to the different bone morphometry and mineralization for specimens (*n* = 2 p/treatment) retrieved at a single time point (10 weeks in vivo). Results indicated a variety of load-transfer ability for the different treatments, highlighting the mechanical adaptation of bone structure in the early stages of bone healing. Although exploratory due to the limited sample size, the findings and analysis reported herein suggest how the combination of microCT and DVC can provide enhanced understanding of the micromechanics of newly formed bone produced in vivo, with the potential to inform further development of novel bone regeneration approaches.

## 1. Introduction

Bone tissue possesses excellent healing capacity as a result of the regenerative growth and remodeling process [1,2]. However, a number of clinical situations, including high-energy trauma, tumor resection, and musculoskeletal diseases, impair the natural bone-healing process, due to the critical size of the defects to bridge [3,4,5]. Common treatments for critical-sized bone defects include bone autografts and allografts [6]. Although autografts are considered the gold standard for promoting bone repair [7,8,9], they present several limitations, including risks of donor site injury, morbidity, and limited availability [10,11]. Bone tissue engineering is therefore seeking more efficient strategies to further regenerate viable bone tissue through a combination of cells, biomaterials, bone active proteins, and drugs [12,13,14]. However, it is important that biomaterials can provide a balanced ability to mechanically support initial scaffolding in the defect site, while encouraging cells to migrate onto and commence the bone healing process. Bone morphogenetic proteins (i.e., BMP-2) have been shown to play a critical role in bone formation and healing, given their capacity to induce osteoblast differentiation [15,16,17]. BMP-2 is a highly osteoinductive growth factor, generally delivered onto an osteoconductive carrier, such as collagen, which provides the structural matrix for bone regeneration [18,19]. However, the supraphysiological doses of BMP-2 associated with the suboptimal delivery systems employed, are associated with heterotopic ossification, osteolysis, and swelling [20], and can increase the incidence of cancer [21]. Therefore, the development of optimal delivery systems of BMPs is an area of intense research, with the aim of providing spatiotemporal control of BMP release in vivo [12,22].

In order to validate and improve bone tissue engineering strategies, it is essential to evaluate the in vivo competence of the newly formed bone induced following applied treatment [23,24]. Critical bone defect animal models have been widely used to provide a robust evaluation of the in vivo response (i.e., new bone formation, remodeling, implant resorption) to regenerative biomaterials [18,25,26,27]. However, a biomechanical evaluation of the newly regenerated bone promoted by different applied treatments and, particularly, the local strain distribution of the engineered tissue is still lacking. This evaluation is of fundamental importance to demonstrate the ability of such tissue engineering approaches to restore new bone with biological and biomechanical properties that are comparable with the native bone. Specifically, understanding the load-bearing capacity and load-transfer mechanisms in relation to the newly formed bone microstructure is essential to fully characterize the mechanical competence of the regenerated tissue within the defect area.

High-resolution X-ray imaging (i.e., micro-computed tomography: microCT) has been extensively used to investigate, in a three-dimensional (3D) manner, the ex vivo implantation site in a non-destructive way [28]. MicroCT imaging allows for a morphological analysis of the regenerated bone tissue [29], as well as the distribution and degree of tissue mineralization, when appropriate densiometric calibration is carried out [30]. Furthermore, when combined with in situ mechanical testing [31] and digital volume correlation (DVC) [32,33], microCT provides a unique tool to investigate the 3D internal deformation of bone down to the tissue level. To date, in situ microCT mechanics in conjunction with DVC have been used to examine 3D full-field displacement and strain in trabecular bone [34,35], cortical bone [36], whole bones [37], and bone–biomaterial systems [38,39,40]. However, to the author’s knowledge, in situ microCT mechanics in conjunction with DVC have never been applied to newly regenerated bone tissue entirely produced in vivo following the implantation of osteoinductive biomaterials in critical-sized bone defects.

This study aims to investigate the 3D full-field strain distribution throughout the apparent elastic regime in newly regenerated bone tissue produced in a critical size bone defect model following the implantation of different osteoinductive biomaterials, including BMP-2 delivery systems, as well as autografts. Bone mineral distribution, morphometry, and load transfer response are evaluated, combining high-resolution microCT, in situ mechanical testing and DVC. Despite the limited number of specimens retrieved at a single time point and evaluated, the findings of this paper provide important insights on the relationships between deformation, microstructure, and mineralization of newly formed bone; hence, improving the description and understanding of the bone regeneration process as a result of different tissue engineering strategies.

## 2. Materials and Methods

### 2.1. Specimen Preparation

Bone defects (9 mm diameter by 10 mm depth) were performed bilaterally in the medial femoral condyles of aged (>5 years) Welsh Upland Ewes (60–75 kg) under Home Office license PPL30/2880. Three different treatments were applied: Autograft, InductOs® (Medtronic BioPharma B.V., Harleen, Netherlands) [41], and Laponite [20,42]. Autograft was prepared intraoperatively from medial femoral condyles. After defect creation, two bone cores (9 mm × 12 mm), were morselized and the graft material was then applied to the defect. InductOs and Laponite consist of a collagen sponge incorporating BMP-2 in a formulation buffer or in a Laponite clay gel, respectively. Briefly, collagen constructs were assembled, adding 22 µL of Medtronic BMP-2 (1.5 mg/mL) to a collagen disc, followed by either 47.1 µL formulation buffer for the InductOs specimens or 47.1 µL of 2.5% Laponite. Two layers of sponge were placed in a mold and a final third BMP-2 loaded collagen sponge was added at the top without InductOs buffer or Laponite. A total of 66 µL of BMP-2 (100 µg) was applied per construct.

Condyles were harvested 10 weeks post implantation and fixed in 4% paraformaldehyde solution in a phosphate buffered solution (PBS) for one week at 4 °C. Cylindrical bone specimens (n = 6, ~5 mm in diameter and ~11 mm in length) were obtained from the bone defect areas by drilling with a trephine blur drill under constant water irrigation. The ends of the cores were trimmed plane and parallel. Specimens were kept in PBS at 4 °C prior to in situ microCT mechanical testing. Pre-testing, brass endcaps were used to embed the ends of the specimens (~2 mm each side) to ensure perpendicularity between the bone cores and the endcaps base. In total, six specimens were prepared (n = 2/treatment).

### 2.2. In Situ Mechanics and MicroCT Imaging

**Figure 1:**
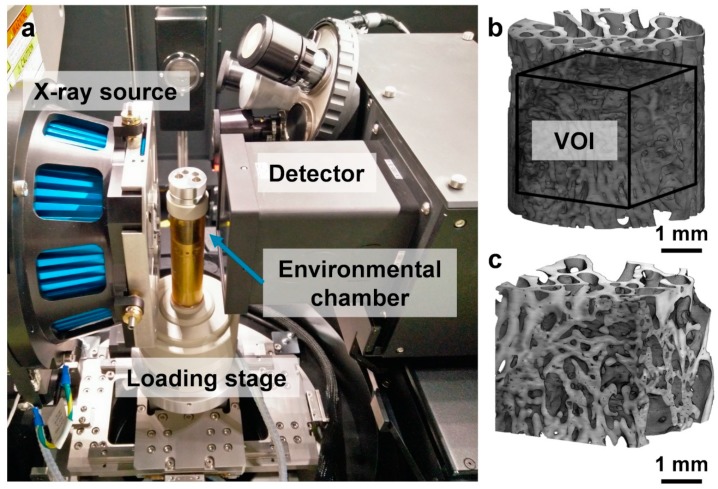
(**a**) Experimental setup for in situ micro-computed tomography (microCT) experiment in the Zeiss Versa 510. Specimens were imaged within an environmental chamber inside the loading stage; (**b**) 3D volume reconstruction of a cylindrical specimen (InductOs#1) and (**c**) a corresponding cubic (3.5 × 3.5 × 3.5 mm^3^) volume of interest (VOI) that was cropped at the center of each specimen.

Specimens were placed within an environmental chamber in a loading device (CT500, Deben Ltd, Suffolk, UK) that was positioned in the chamber of a high-resolution 3D X-ray microscope (Versa 510, Zeiss, Pleasanton, CA, USA) (Figure 1a). Uniaxial step-wise compression was carried out in displacement control at a constant cross-head speed of 0.2 mm/min. First, a small preload (~2 N) was applied to ensure good end contact prior to testing, followed by step-wise compression at three different levels of apparent compression (1%, 2%, and 3%). The force-displacement recordings from the loading device were used to calculate the stiffness of the specimens. A linear regression equation (coefficient of determination R^2^ > 0.997) was used to fit the experimental data between 0.8% and 2% compression and the stiffness was determined from the slope of that line. MicroCT images were acquired (60 keV, 5 W, 5 µm voxel size, 10 s exposure time, 1800 projections) at each compression step after two repeated scans in the preload configuration for DVC error analysis. Image reconstruction was performed via the manufacturer’s software (TXM Reconstructor, Zeiss, Pleasanton, CA, USA). In total, five tomographic datasets were acquired for each specimen.

### 2.3. Mineral Density Distribution

A densiometric calibration phantom (microCT-HA, QRM, Moehrendorf, Germany) containing five insertions with hydroxyapatite (HA) concentrations (1200 mgHA/cm^3^, 800 mgHA/cm^3^, 200 mgHA/cm^3^, 50 mgHA/cm^3^, 0 mgHA/cm^3^) was imaged under identical experimental conditions and used to calibrate the microCT grey-scale values of the first acquired tomogram for each specimen into tissue mineral density (TMD). A cubic volume of interest (VOI) with side lengths of 700 voxels (3.5 mm^3^) was cropped from the center of the scaled images (Figure 1b,c) and the average and peak TMD (Avg-TMD and Peak-TMD) were calculated from the calibrated histograms of each specimen.

### 2.4. Image Post-Processing

Following image reconstruction, the microCT datasets were rigidly aligned using the first acquired tomogram as reference. The rigid registration was based on the sum of squares differences as a similarity measurement. The images were then denoised by applying a non-local means filter (σ = 10) (Figure 1c) and masked by setting the grey-scale values corresponding to the non-mineralized tissue voxels to zero (i.e., bone marrow). A binary image was first created by applying a global thresholding based on Otsu’s algorithm [43], followed by a “purify” analysis in BoneJ [44] plugin for Fiji [45], which locates all particles in 3D and removes all but the largest foreground (bone tissue) and background (bone marrow) particles. Masked images were obtained by multiplying the filtered image to the binary image.

### 2.5. Morphometric Analysis

Morphometric analysis was performed using the BoneJ [44] plugin. The same cubic VOI as for the mineral density distribution (Figure 1c) was cropped from the binary images and bone volume fraction (BV/TV) was computed. Due to the irregular bone microarchitecture induced by the different treatments, trabecular thickness (Tb.Th) and trabecular spacing (Tb.Sp) were computed in smaller sub-VOIs (1.75 mm^3^). Data were subsequently screened using Peirce’s criterion [46] to identify outliers and to remove large voids or dense bone regions from the computation. Following the removal of outliers, mean Tb.Th and Tb.Sp were calculated for each specimen.

### 2.6. Digital Volume Correlation

DVC (DaVis v8.4, LaVision, Göttingen, Germany) was carried out to evaluate the 3D full-field strain distribution in the newly regenerated bone tissue throughout the apparent elastic regime. DaVis software is based on a local approach [47] of deformable registration, which has been extensively used in bone mechanics [35,37,40,48]. The operating principles of the algorithm are detailed elsewhere [49]. DVC was applied to the masked images (entire cylinders) to avoid possible artefacts in regions with insufficient grey-scale pattern (i.e., bone marrow). A multi-pass scheme with a final sub-volume of 40 voxels (200 µm), reached via predictor passes using sub-volumes of 56, 48, and 44 voxels, was used (Supplementary Figure S1). Furthermore, sub-volumes with a correlation coefficient below 0.6 were removed from the resultant vectors. Errors on the DVC-computed displacement (zero-strain test) did not exceed 2 µm (Supplementary Figure S2), whereas the mean absolute error (MAER) and the standard deviation of the error (SDER) of the strain components [50] were found to be ~530 µε and 160 µε, respectively (Supplementary Figure S1). To evaluate the 3D full-field strain distribution over time in relation to the deformation induced by the compressive applied load, the third principal strain (ε_p3_) for each loading step was computed within the bone volume in the VOI (Figure 1b) previously described, after a bi-cubic interpolation.

### 2.7. Statistical Analysis

Quantitative analysis on local compressive strains were reported as mean, median, and inter-quartile range between 25th and 75th percentile of the data on violin plots. Two-tailed Pearson correlation analysis was performed between microstructural parameters (i.e., TMD, BV/TV, and Tb.Th) and mechanical properties (i.e., stiffness and mean local compressive strain) for all six specimens. Additionally, two-tailed Pearson correlation analysis was done between the mean local strains at the most (20% of the BV highly strained) and least (remaining volume) strained regions at the maximum applied compression and microstructural parameters. Both regions within the same specimens were considered related, thus the non-parametric Wilcoxon signed rank test (α = 0.05) was used to compare strain magnitude, trabecular thickness, and tissue mineral densities. All statistical analyses were done using Matlab software (Matlab R2017a, The MathWorks Inc., Natick, MA, USA).

## 3. Results

### 3.1. In Situ Mechanical Testing and MicroCT Imaging

**Figure 2:**
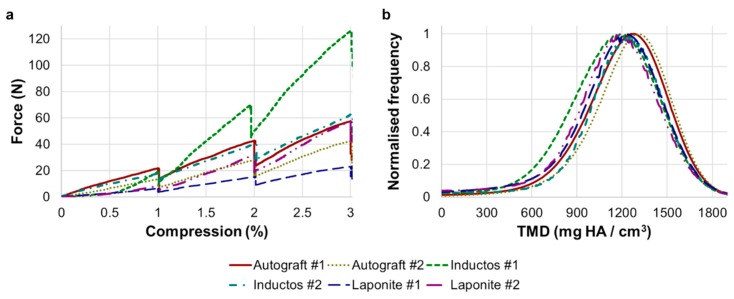
(**a**) Force–compression curves for the six specimens. The force shows a drop at the end of each step of compression, which corresponds to the time the specimen was allowed to settle (~30 min) prior to image acquisition (~5 h). An initial toe region was observed in InductOS #1 and Laponite #2, mainly dependent on the initial lack of co-planarity between the ends of the specimens; (**b**) Normalized tissue mineral density (TMD) histograms for the six analyzed specimens showed minimal differences in peak values and distribution.

The in situ mechanical tests (Figure 2a) demonstrated the distinct response of each specimen under the same applied compression, with stiffness ranging from 131.9 N/mm for Laponite #1 to 547.6 N/mm for InductOS #2 (Table 1). Force–compression curves exhibited a monotonic profile that was observed to be, in essence, linear during the three loading steps. The TMD distribution (Figure 2b) in the VOI of the specimens showed little difference, with a mean Avg-TMD of 1168.5 mgHA/cm^3^ (±50.4 mgHA/cm^3^) and mean Peak-TMD of 1236.4 mgHA/cm^3^ (±47.4 mgHA/cm^3^) (Table 1).

**Figure 3:**
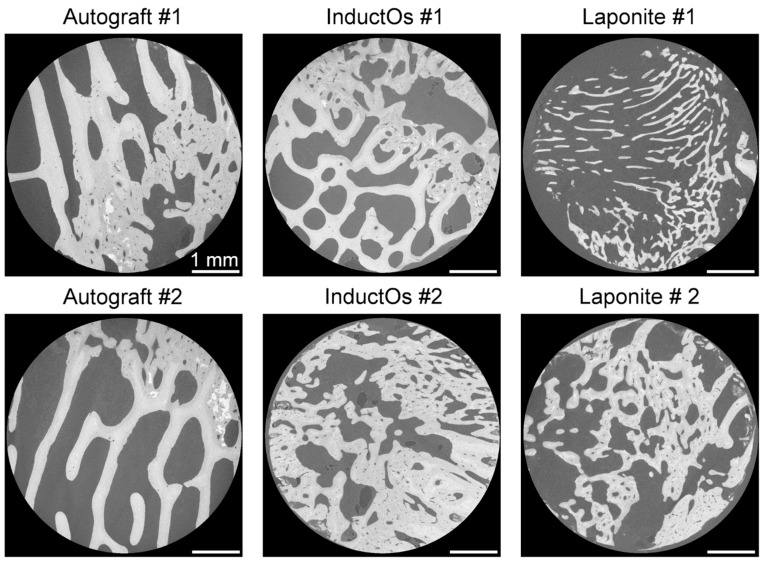
MicroCT cross-sections through the six analyzed specimens, showing differences in microstructure and mineralization. Large agglomerates of mineral (white) were identified in Autograft specimens. Laponite #1 showed considerably thinner trabeculae. Large areas of woven bone were present in InductOs and Laponite #2 specimens. Scale bars = 1 mm.

High-resolution microCT images (Figure 3) provided visualization of the characteristic microstructure and mineral distribution in the newly formed bone induced by the different treatments. Greater remodeled bone trabeculae were observed in Autograft specimens in comparison to other groups, with the presence of highly mineralized regions (lightest grey) within the newly formed bone. InductOs specimens presented the highest BV/TV, above 50% (Table 1), with dense areas of woven bone. InductOs #1 was able to regenerate trabeculae with comparable thickness to Autograft specimens (~250 µm), whereas thinner trabeculae (~150 µm) were observed in InductOs #2 (Table 1). The thinnest trabeculae (below 150 µm) were identified in Laponite specimens, where larger voids were found in Laponite #1, which was unable to entirely bridge the defect site, compared to Laponite #2 at a 10-week point of retrieval.

Overall, large variations were observed in both the mechanical and microstructural parameters among all specimens. Moderate correlations were found between the stiffness, TMD, and BV/TV, although not significant, whereas the stiffness correlated poorly with the Tb.Th (Supplementary Figure S3).

### 3.2. Digital Volume Correlation

**Figure 4:**
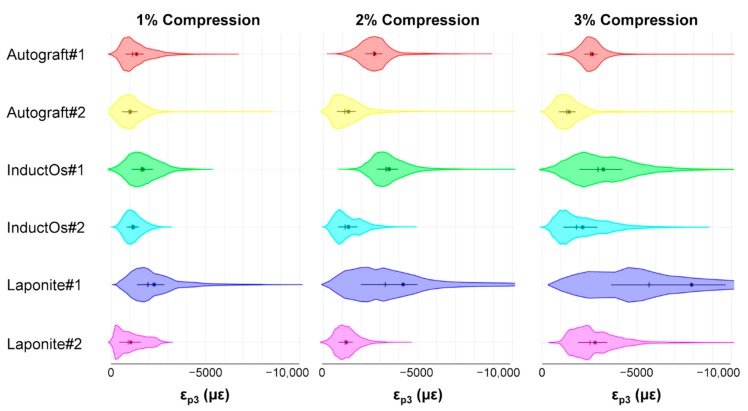
Violin plots showing the third principal strain distribution (ε_p3_) in the VOI of the six specimens at each compression step smoothed by a kernel density function. Median (+) and mean (*) values are indicated in each plot. Horizontal lines show the inter-quartile range (IQR, between 25th and 75th percentile of the data).

The computed local compressive strain (ε_p3_) accumulation after each loading step is shown in Figure 4. Autograft specimens showed small differences between mean and median values, whose magnitudes ranged between 1000 µε at 1% apparent compression and 2500 µε at 3%, with highly homogeneous distributions and thin tails. Higher strain accumulation was observed for InductOs and Laponite specimens, which showed a broader local strain distribution. This was more evident at 3% compression, where mean and median values differed considerably, and large inter-quartile ranges were found. The highest compressive strains were observed in Laponite #1, with mean strain magnitudes varying from 2000 µε at 1% apparent compression to 8000 µε at 3%, whereas mean strain magnitudes never exceeded 3500 µε for the other specimens.

**Figure 5:**
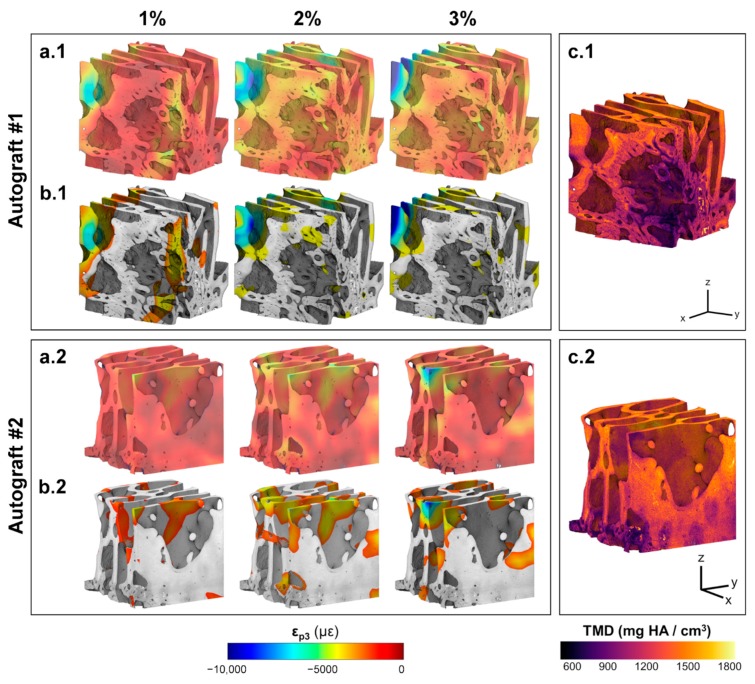
(**a**)(**a.1**,**a.2**) 3D full-field third principal strain distribution (ε_p3_) at each compression step for the VOI of Autograft specimens and (**b**)(**b.1**,**b.2**) corresponding 20% of the bone volume fraction (BV/TV) that was highly strained. An increase and redistribution in the accumulated strain was observed following consecutive compression steps. (**c**)(**c.1**,**c.2**) Tissue mineral density distribution in the VOI of each specimen. The greater remodeled trabeculae showed higher mineralization compared to newly formed bone tissue in both specimens.

**Figure 6:**
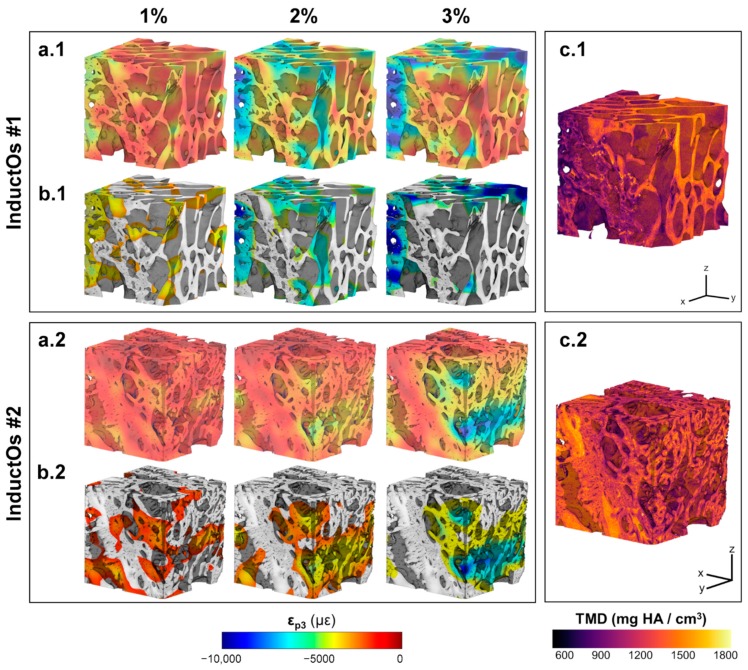
(**a**)(**a.1**,**a.2**) 3D full-field third principal strain distribution (ε_p3_) at each compression step for the VOI of InductOs specimens, and (**b**)(**b.1**,**b.2**) corresponding 20% of the bone volume fraction (BV/TV) that was highly strained. An increase and redistribution in the accumulated strain was observed after consecutive compression steps. (**c**)(**c.1**,**c.2**) Tissue mineral density (TMD) distribution in the VOI of each specimen. The more remodeled trabeculae showed higher mineralization compared to the newly formed bone tissue in InductOs #1. Highly mineralized areas were identified in the woven bone for InductOs #2.

**Figure 7:**
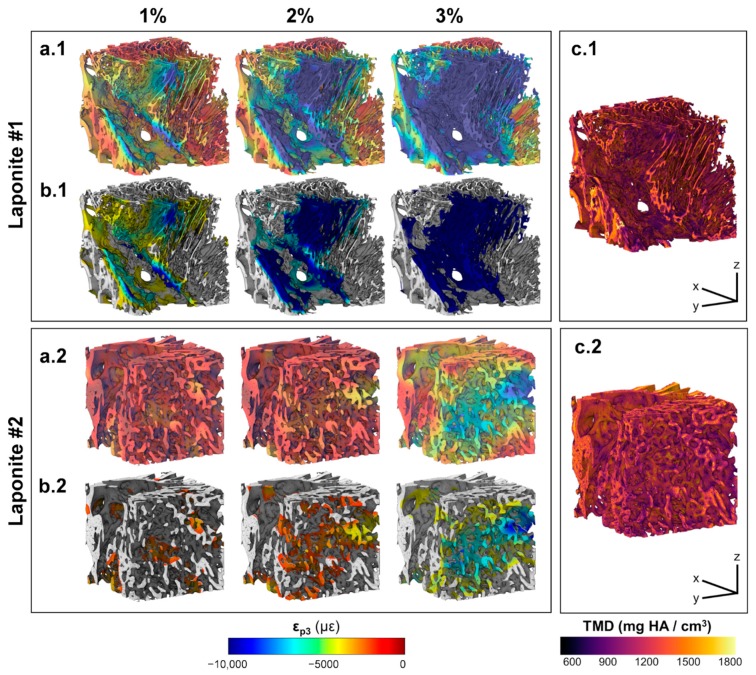
(**a**)(**a.1**,**a.2**) 3D full-field third principal strain distribution (ε_p3_) at each compression step for the VOI of Laponite specimens, and (**b**)(**b.1**,**b.2**) corresponding 20% of the bone volume fraction (BV/TV) that was highly strained. An increase and redistribution in the accumulated strain is observed after consecutive compression steps. (**c**)(**c.1**,**c.2**) Tissue mineral density distribution in the VOI of each specimen. Highly mineralized regions were found within the woven bone, whereas the newly formed thin trabeculae showed lower mineralization.

The 3D full-field ε_p3_ in Autograft specimens (Figure 5a) revealed local strain accumulation with increasing compression levels. Areas of high strain concentration (Figure 5b) were predominantly within the more remodeled trabeculae from the first compressive step, with strain magnitudes exceeding −8000 µε in some regions at 3% compression. Newly formed bone regions showed less mineralization (Figure 5c) compared to the greater remodeled trabecular bone, however this was not related to higher deformation. Strain accumulation within the InductOs specimens (Figure 6a) was observed to be more progressive than for Autograft specimens, indicating larger differences at each compressive level. Highly strained areas were typically observed in the newly formed bone (woven) for InductOs #1 and in the newly formed trabeculae in InductOs #2 (Figure 6b), which showed less mineralization (Figure 6c). The Laponite #1 specimen (Figure 7a.1) displayed a marked and abrupt strain accumulation compared to Laponite #2 (Figure 7a.2), with areas with strain magnitude exceeding 10,000 µε from the first compression step (Figure 7b.2). The ε_p3_ in Laponite #2 was largely homogeneous after 1% and 2% of applied compression; however, larger strain values were found at 3% compression. Both specimens experienced higher strains in the newly formed thin trabeculae (Figure 7b), which also matched the lower mineralized regions (Figure 7c). TMD was found to be higher in the new bone.

Local compressive strain among all specimens correlated poorly with the TMD, but moderately with the BV/TV and overall thickness (Supplementary Figure S3). Highly strained regions (mean of all the specimens, n = 6) at the maximum load (3% apparent compression) presented significantly lower thickness compared to the less strained areas (mean of all the specimens, n = 6), although no significant differences were observed in the Avg-TMD between both regions (Supplementary Figure S4).

## 4. Discussion

The main aim of this study was to evaluate the load-bearing capacity of newly regenerated bone tissue following the action of different osteoinductive biomaterials implanted in a large animal critical bone defect. High-resolution X-ray imaging in combination with in situ mechanical testing and DVC was used to obtain the full-field strain accumulated in the tissue during step-wise compression within the apparent elastic region. This was further analyzed in relation to bone microarchitecture and tissue mineral density, providing a qualitative assessment on the local mechanical properties of regenerated bone.

The distinct treatments applied to the bone defects had a clear influence on the microstructure of the newly regenerated tissue (Figure 3) and, consequently, on the mechanical properties at both apparent and tissue level. Ten weeks post-implantation was observed to be insufficient time to completely remodel bone within the defect; thus, dense areas of woven bone were still visible in all specimens. However, autograft induced a more regular bone regeneration, as evidenced by the greater remodeled trabeculae, compared to the BMP-2 treated defects. Both InductOs and Laponite specimens showed a considerably irregular microarchitecture and the presence of large voids within the structure. In vivo, collagen degrades rapidly, resulting in the development of voids within the new bone matrix. This is mainly due to the inability of collagen to provide structural support for cell migration in adequate time [51,52]. InductOS specimens induced the greatest bone formation (Table 1), although large regions of woven bone were still observed. Conversely, Laponite induced the thinnest trabeculae, but less woven bone. Trabecular thickness and spacing of all the specimens were measured in smaller sub-VOIs and examined for possible outliers [46] to avoid misleading data as a consequence of compact woven regions and empty voids. As a result, outliers were removed from the data and the presented values (Table 1) are representative of the trabecular-like structure of each specimen.

The different microarchitecture induced in vivo in the bone defects clearly influenced their apparent mechanics (Figure 2a). It is known that the elastic behavior of trabecular bone depends on the loading direction [53,54,55,56], anatomical site [57,58,59], and size of the specimen [60,61,62]. This study did not show a significant correlation between the measured stiffness and any microstructural parameters, although the stiffness moderately correlated with the BV/TV (R = 0.776, Supplementary Figure S3). However, it must be noted that specimens tested were not fully remodeled into trabecular bone at the time of extraction. Furthermore, the specimens were not cored and tested along the principal direction, mainly due to the experimental difficulties related to the bone defect size and location of the newly formed bone within specimens. While the microstructure and stiffness of the analyzed specimens clearly differed, negligible changes were observed in their TMD distribution (Figure 2b). Higher mineralization was found in the greater remodeled trabeculae of the Autograft (Figure 5c) and InductOs #1 (Figure 6c.1) specimens compared to the less remodeled and thinner trabeculae in InductOs #2 (Figure 6c.2) and Laponite (Figure 7c) specimens.

The DVC-computed full-field strain in the specimens allowed for characterization of the load transfer ability of the newly regenerated bone structures. Even though applied loads at each compression step did not correspond to any physiological load in vivo, specimens were predominantly tested in their apparent elastic regime. This provided enhanced insight on the load-bearing capacity of such bone structures away from failure. A more homogeneous strain distribution was observed for Autograft specimens (Figure 5a) during the three compression steps, where the bone tissue experienced strains mainly within physiological strain values (magnitude below 3200 µε [63]). This is consistent with previous DVC data obtained for untreated trabecular bone specimens with similar dimensions to the one used in the current study and from the same animal model, where the mean compressive strain magnitude at 3% of compression did not exceed 1000 µε [40]. For InductOs #2 (Figure 6a.1) and Laponite #2 (Figure 7a.1), during the first two compression steps, strains were typically below 3000 µε in magnitude, and only higher strain concentrations were observed after 3% compression in the newly formed trabeculae, with strain values approaching the yielding (~10,000 µε in compression [64]). In contrast, full-field strain distribution in InductOs #1 and Laponite #1 showed the inefficiency of both structures to transfer the applied load. As a result, highly-strained regions (above yielding) were observed and further increased since the early stages of compression, 1% for Laponite #1 (Figure 7a.2) and 2% for InductOs #1 (Figure 6a.2). For instance, 22% of Laponite #1 volume exceeded yielding at 3% apparent compression. Local mechanics appeared to be more influenced by the microstructure than the degree of mineralization (Supplementary Figure S3). In particular, local thickness moderately correlated (R = 0.660, Supplementary Figure S3) with local compressive strains, whereas correlation between thickness and apparent mechanics (i.e., stiffness) was poor (R = 0.269, Supplementary Figure S3).

The identification of highly strained regions further improved the understanding of failure development in such structures and how the regeneration process, with changes in bone microarchitecture and mineralization, is optimized to bear externally applied loads. Highly strained regions were predominantly localized in the newly regenerated trabeculae (thinner regions) in all the specimens, being on average 18% thinner, independent of mineralization (Supplementary Figure S4). This suggests that from the earliest stages of bone formation, mechanical strain is stimulating and promoting bone structural adaptation to withstand external loads [65], irrespective of the specific osteoinductive treatment. In fact, the enhanced mineralized regions of the BMP-2 treated specimens (Figure 6 and Figure 7) appeared to act as a shielding mechanism, driving the load towards the trabecular-like structure, which is consequently adapted to the external mechanical demands in vivo. It is well known that the mechanical environment highly influences the pathways through which bone healing occurs [66,67]. However, determining the precise strains that tissues are experiencing in vivo remains a significant challenge [68] and most research to date has focused on in silico studies [69].

A number of caveats must be considered within this study. The limited quantity of newly formed bone within the defects resulted in a limited sample size (two specimens per treatment) and the inability to extract the specimens in the principal direction of the newly formed trabeculae. However, in situ microCT and DVC experiments are typically demanding in terms of time and computational cost, which makes the analysis more qualitative than quantitative, as previously proposed in another ex vivo analysis of bone-biomaterial systems [40]. Also, the current study did not aim to evaluate the efficacy of one treatment over the others, but to investigate the local mechanical competence of regenerated bone tissue entirely induced in vivo by the different biomaterials and explore the differences in both mechanics and microstructure at a single time point. These were large animal ovine studies and large variations in both mechanical and structural parameters were evidenced among the samples. Unfortunately, the maintenance cost of large animal models and compliance with the replacement, reduction, and refinement of animals in research inevitably reduce numerosity with the ability to investigate multiple time points [40]. Nevertheless, the dissimilarities of these newly formed bone structures produced evidence of the relationship between apparent and local strains and the irregular microarchitecture induced in vivo during the remodeling phase of bone regeneration. Furthermore, these findings have the potential to inform numerical models on bone adaptation, enabling predictions of long-term bone healing following different tissue engineering approaches, as well as the determination of material properties and the relative contribution of the different tissues (i.e., both hard and soft tissues) to the local mechanical behavior of these complex structures. While it could be argued that the mechanics of bone tissue may be altered due to the fixation process, it should be noted that the specimens were left in fixative for only one week and testing was undertaken in the apparent elastic regime. It has been shown that fixation for up to four weeks does not alter the compressive properties of bone [70], and appears not to influence the elastic properties of bone [71]. Additionally, the calibration phantom was not included with each specimen to be imaged; therefore, the intrinsic fluctuations of the microCT system (i.e., tube voltage level, current), which could potentially affect the grey-scale levels, were not accounted for [30]. However, the purpose of the presented calibration was to better understand how high and low mineralized regions of bone correlated with newly formed bone, as opposed to a precise determination of the density measures of each specimen.

## 5. Conclusions

The combination of high-resolution X-ray microCT, in situ mechanics and DVC was used to qualitatively characterize the full-field strain distribution and load-bearing capacity of newly formed bone induced in vivo in a large animal critical size bone defect, following different treatments. The local strain development in the tissue was evaluated in relation to bone microarchitecture and mineralization, providing new information on the micromechanics of regenerated bone. Despite the limited number of specimens retrieved (n = 2 p/treatment) at a single time point (10 weeks in vivo) and evaluated, DVC-computed strain distributions seemed to indicate that the regenerated bone structure is mechanically adapted to bear external loads from the early remodeling stages of the bone reparation cascade. The results of this study provide an important step towards understanding the load transfer ability and local mechanical properties of newly formed bone in vivo. The experimental techniques presented herein in unravelling local mechanics in regenerated tissue can be further applied to understand targeted biomaterial action in vivo. Such information is crucial to facilitate the development of novel regeneration strategies designed to promote tissue with comparable biomechanical quality to the native bone.

## Figures and Tables

**Table 1:** Overview of the mechanical, mineral, and morphological parameters of the analyzed specimens. For each specimen, stiffness, average tissue mineral density (Avg-TMD), peak tissue mineral density (Peak-TMD), bone volume fraction (BV/TV), trabecular thickness (Tb.Th), and trabecular spacing (Tb.Sp) are detailed.

Specimen	Stiffness (N/mm)	Avg-TMD (mgHA/cm^3^)	Peak-TMD (mgHA/cm^3^)	BV/TV (%)	Tb.Th (µm)	Tb.Sp (µm)
Autograft #1	249.3	1207.8	1279.5	46.1	222 ± 71	484 ± 168
Autograft #2	212.1	1247.9	1314.8	33.9	267 ± 72	736 ± 246
InductOs #1	547.6	1107.2	1185.4	52.2	233 ± 68	477 ± 183
InductOs #2	322.6	1183.1	1232.5	54.6	147 ± 41	338 ± 198
Laponite #1	131.9	1152.3	1220.7	22.4	107 ± 47	466 ± 282
Laponite #2	363.0	1112.5	1185.4	45.4	141 ± 35	339 ± 189

## Supplementary Materials

The following are available online at https://www.mdpi.com/1996-1944/13/1/168/s1, Figure S1: Strain uncertainties analysis; Figure S2: Random errors analysis; Figure S3: Correlations between mechanical and microstructural parameters; Figure S4: Thickness and mineralization differences between regions highly and lowly strained.
