# Supplementary material for: Exploratory Full-Field Strain Analysis of Regenerated Bone Tissue from Osteoinductive Biomaterials
Source: Materials (Basel). 2020 Jan 1;13(1):168. doi: 10.3390/ma13010168 (PMC6981952; doi:10.3390/ma13010168)
Supplement: Supplementary file 1 [file materials-13-00168-s001.pdf]

materials

MDPI

Article

Exploratory Full-Field Strain Analysis of Regenerated Bone Tissue from Osteoinductive Biomaterials

Marta Peña Fernández [1], Cameron Black [2], Jon Dawson [2], David Gibbs [2,3], Janos Kanczler [2], Richard O.C. Oreffo [2] and Gianluca Tozzi [1,*]

1 School of Mechanical and Design Engineering, University of Portsmouth, Portsmouth PO1 3DJ, UK
2 Bone & Joint Research Group, Centre for Human Development Stem Cells and Regeneration, Faculty of Medicine, University of Southampton, Southampton SO16 6YD, UK
2 School of Maritime Science and Engineering, Solent University, Southampton SO14 0YN, UK
* Correspondence: Gianluca.tozzi@port.ac.uk; Tel.: +44-(0)-239284-2514

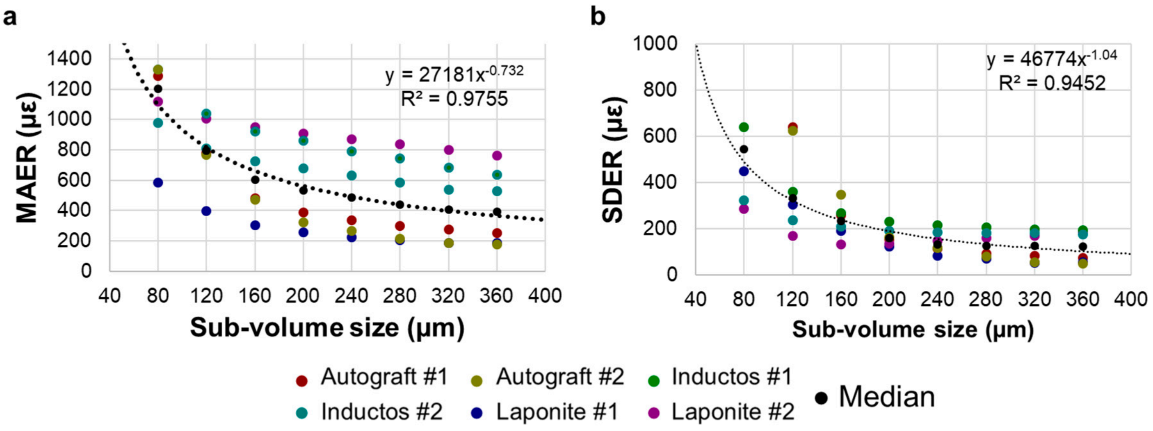

Figure S1. Strain uncertainties analysis. Relationship between (**a**) mean absolute strain error (MAER) and (**b**) standard deviation of the error (SDER) of the strain components with the sub-volume size for the specimens (n = 6). Median values are also reported.

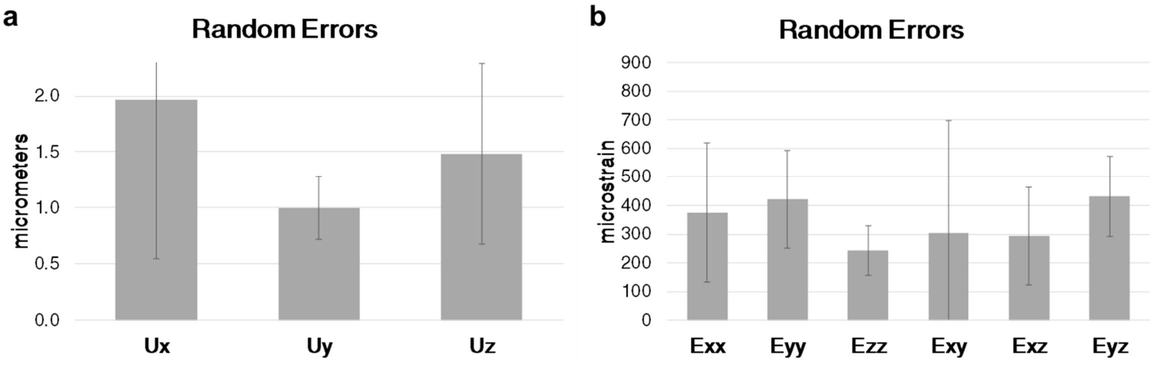

Figure S2. Random errors analysis. (**a**) Displacement random errors and (**b**) random errors of each strain component for the analysed specimens for a final sub-volume size of 40 voxels. Bars represent the median value whereas error bars represent the standard deviation accounting for the total number of specimens (n = 6).

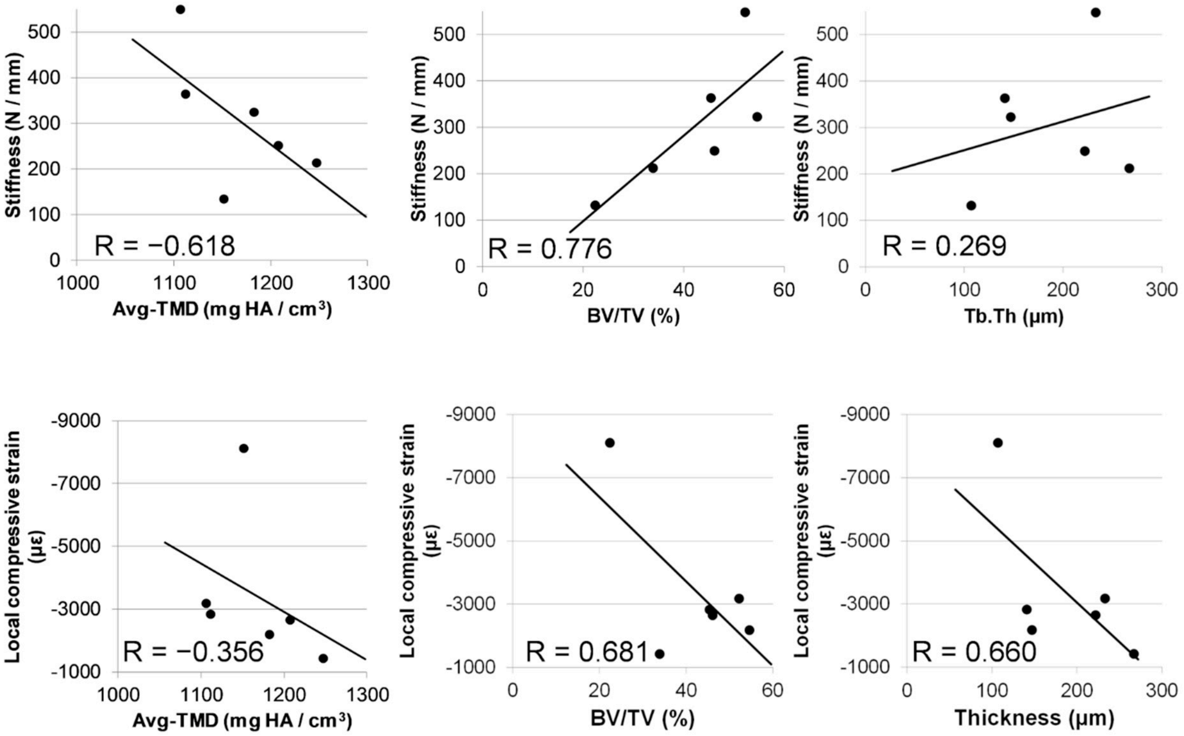

Figure S3. Correlations between mechanical and microstructural parameters for the total number of specimens (n = 6). Scatterplots showing linear correlations at (top) apparent level (stiffness, average TMD, BV/TV and Tb.Th) and (bottom) tissue level (local compressive strain, TMD, BV/TV and local thickness). Pearson correlation coefficients (R) are indicated.

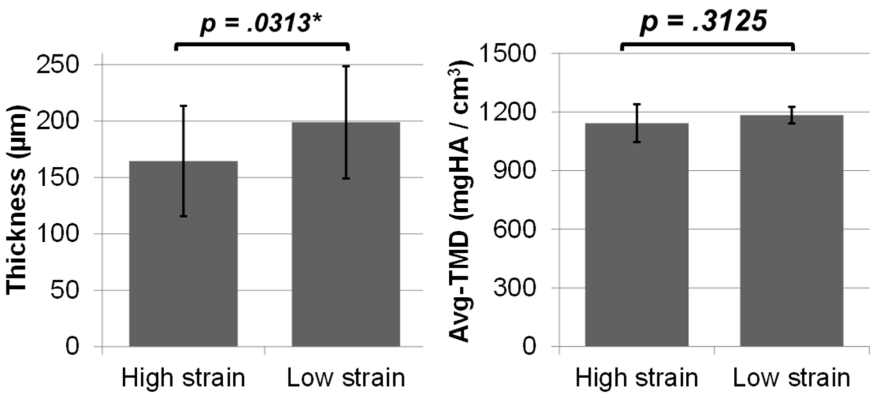

Figure S4. Thickness and mineralization differences between regions highly and lowly strained (mean of all the specimens, n = 6). Local thickness (left) and average TMD (right) of the highly and lowly strain regions at the maximum apparent compression (3%). p-values indicate significant difference based on Wilcoxon signed rank tests for paired samples. Mean ± standard deviation among the six specimens are shown.

© 2020 by the authors. Licensee MDPI, Basel, Switzerland. This article is an open access article distributed under the terms and conditions of the Creative Commons Attribution (CC BY) license (http://creativecommons.org/licenses/by/4.0/).
